# Durability of Olyset campaign nets distributed between 2009 and 2011 in eight districts of Tanzania

**DOI:** 10.1186/s12936-016-1225-6

**Published:** 2016-03-18

**Authors:** Dennis J. Massue, Sarah J. Moore, Zawadi D. Mageni, Jason D. Moore, John Bradley, Olivier Pigeon, Erasto J. Maziba, Renata Mandike, Karen Kramer, William N. Kisinza, Hans J. Overgaard, Lena M. Lorenz

**Affiliations:** Epidemiology and Public Health Department, Swiss Institute of Tropical and Public Health, Soccinstrase 57, 4002 Basel, Switzerland; University of Basel, Petersplatz 1, 4003 Basel, Switzerland; National Institute for Medical Research, Amani Research Centre, Muheza, P. O. Box 81, Tanga, Tanzania; Ifakara Health Institute, Bagamoyo Research and Training Centre, Bagamoyo, P.O. Box 74, Pwani, Tanzania; Department of Disease Control, Faculty of Infectious and Tropical Diseases, London School of Hygiene and Tropical Medicine, London, WC1E 7HT UK; MRC Tropical Epidemiology Group, London School of Hygiene and Tropical Medicine, London, WC1E 7HT UK; Plant Protection Products and Biocides Physico-chemistry and Residues Unit, Agriculture and Natural Environment Department, Walloon Agricultural Research Centre (CRA-W), Gembloux, Belgium; National Malaria Control Programme, Dar Es Salaam, Tanzania; Institut de Recherche pour le Développement (IRD), MIVEGEC, Montpellier, France; Department of Entomology, Kasetsart University, Bangkok, Thailand; Department of Mathematical Sciences and Technology, Norwegian University of Life Sciences, Ås, Norway

**Keywords:** Durability, LLINs, Olyset, Universal coverage campaign, Attrition, Physical integrity, Chemical content, Bio-efficacy, Tanzania

## Abstract

**Background:**

Long-lasting insecticidal nets (LLINs) are the first line choice for malaria vector control in sub-Saharan Africa, with most countries adopting universal coverage campaigns. However, there is only limited information on LLIN durability under user conditions. Therefore, this study aimed to assess the durability of Olyset^®^ LLINs distributed during campaigns between 2009 and 2011 in Tanzania.

**Methods:**

A retrospective field survey was conducted in eight districts in Tanzania mainland to assess the durability of Olyset campaign nets. Household questionnaires were used to assess attrition, i.e. net loss. All nets remaining in households were collected. A sub-sample of 198 Olyset campaign nets was examined for bio-efficacy against *Anopheles gambiae s.s.* mosquitoes, permethrin content and physical integrity following standard World Health Organization (WHO) methods.

**Results:**

Of 6067 campaign nets reported to have been received between 2009 and 2011, 35 % (2145 nets) were no longer present. Most of those nets had been discarded (84 %) mainly because they were too torn (94 %). Of the 198 sub-sampled Olyset LLINs, 61 % were still in serviceable physical condition sufficient to provide personal protection while 39 % were in unserviceable physical condition according to WHO proportionate Hole Index (pHI). More than 96 % (116/120) of nets in serviceable condition passed WHO bioefficacy criteria while all nets in unserviceable condition passed WHO bioefficacy criteria. Overall mean permethrin content was 16.5 g/kg (95 % CI 16.2–16.9) with 78 % of the sub-sampled nets retaining recommended permethrin content regardless of their age or physical condition. Nets aged 4 years and above had a mean permethrin content of 14 g/kg (95 % CI 12.0–16.0). The only statistically significant predictor of reduced physical net integrity was rats in the house.

**Conclusions:**

Two-to-four years after a mass campaign, only 39 % of distributed nets remain both present and in serviceable physical condition, a functional survival considerably below WHO assumptions of 50 % survival of a ‘three-year’ net. However, the majority of nets still retained substantial levels of permethrin and could still be bio-chemically useful against mosquitoes if their holes were repaired, adding evidence to the value of net care and repair campaigns.

## Background

Long-lasting insecticidal nets (LLINs) have significantly contributed to the success of malaria control in malaria-endemic countries in Africa [1]. In Tanzania in particular, mosquito nets contributed to a 45 % reduction in all-cause mortality in children less than 5 years of age from 146/1000 live births in 1999 to the recent level of 81/1000 live births in 2010 [2]. Since 2009, two LLIN mass distribution campaigns have been implemented in Tanzania, namely the under-five catch-up campaign (U5CC), which provided Olyset^®^ nets to all children under the age of five between 2009 and 2010 [3], and the universal coverage campaign (UCC), between 2010 and 2011 [4], which provided Olyset nets for all sleeping spaces that had not been previously covered during the U5CC campaign. As recommended by the World Health Organization (WHO) [5], LLINs are expected to provide both personal and community protection resulting in a decline in malaria transmission. In addition to the mass distribution campaigns described above, two continuous distribution strategies have been implemented in Tanzania. The Tanzania National Voucher Scheme (TNVS) from 2004 to 2014 provided pregnant women and infants with LLINs at a greatly reduced price [6]. The currently ongoing annual School Net Programme (SNP) in the Southern zone provides every school child in specific grades one free LLIN for distribution to their households [7]. In addition, a universal replacement campaign (URC) is currently ongoing in 2015 and 2016, and is expected to provide 22 million nets to all households in Tanzania not covered by the SNP. All these distribution campaigns aim to reduce malaria transmission in the country through sustainable distribution mechanisms. Since LLINs have a limited serviceable life through loss of chemical insecticide and physical damage, net replacement campaigns are necessary to maintain high coverage, and the timing of these campaigns is of crucial importance.

The useful life of LLINs depends on properties of the net including physical integrity and persistence of insecticide and is not simply a matter of how long the net remains in the house [8]. Durability of LLINs is affected by variation in physical wear, which in turn depends on environmental and social factors like climate, type of sleeping space, presence of rodents or other animals, frequency of use and washing of nets; all of which vary between locations and populations [9–11]. This means that Tanzania’s management decisions regarding LLIN replacement should be based on local LLIN performance data [12]. Information on the durability of different LLIN brands under user conditions will help malaria control programmes by providing information needed to plan the timing of future net replacement campaigns, and the procurement of the most durable LLIN for a country. In addition, information on appropriate net use and care (through improved behaviour change communication) might help to prolong the life of LLINs and reduce the costs of procurement and distribution [13, 14].

In Tanzania, the first choice of LLINs has historically been Olyset^®^ nets, developed by Sumitomo in Japan and manufactured by A–Z Textile Mills Limited in Arusha, Tanzania. Olyset nets, made from 150 denier polyethylene material with permethrin incorporated in the yarn, were the first LLINs to receive the full recommendation of WHOPES in October 2001 for use in prevention and control of malaria [15]. This study aimed to assess the durability of Olyset campaign nets (old knitting pattern) distributed between 2009 and 2011 in eight districts in Tanzania by measuring attrition (net loss), biological efficacy against anopheline mosquitoes (blood feeding inhibition and mortality), chemical content (amount of active ingredient) and physical integrity (number of holes and resulting physical condition of nets).

## Methods

### Study areas

**Fig. 1:**
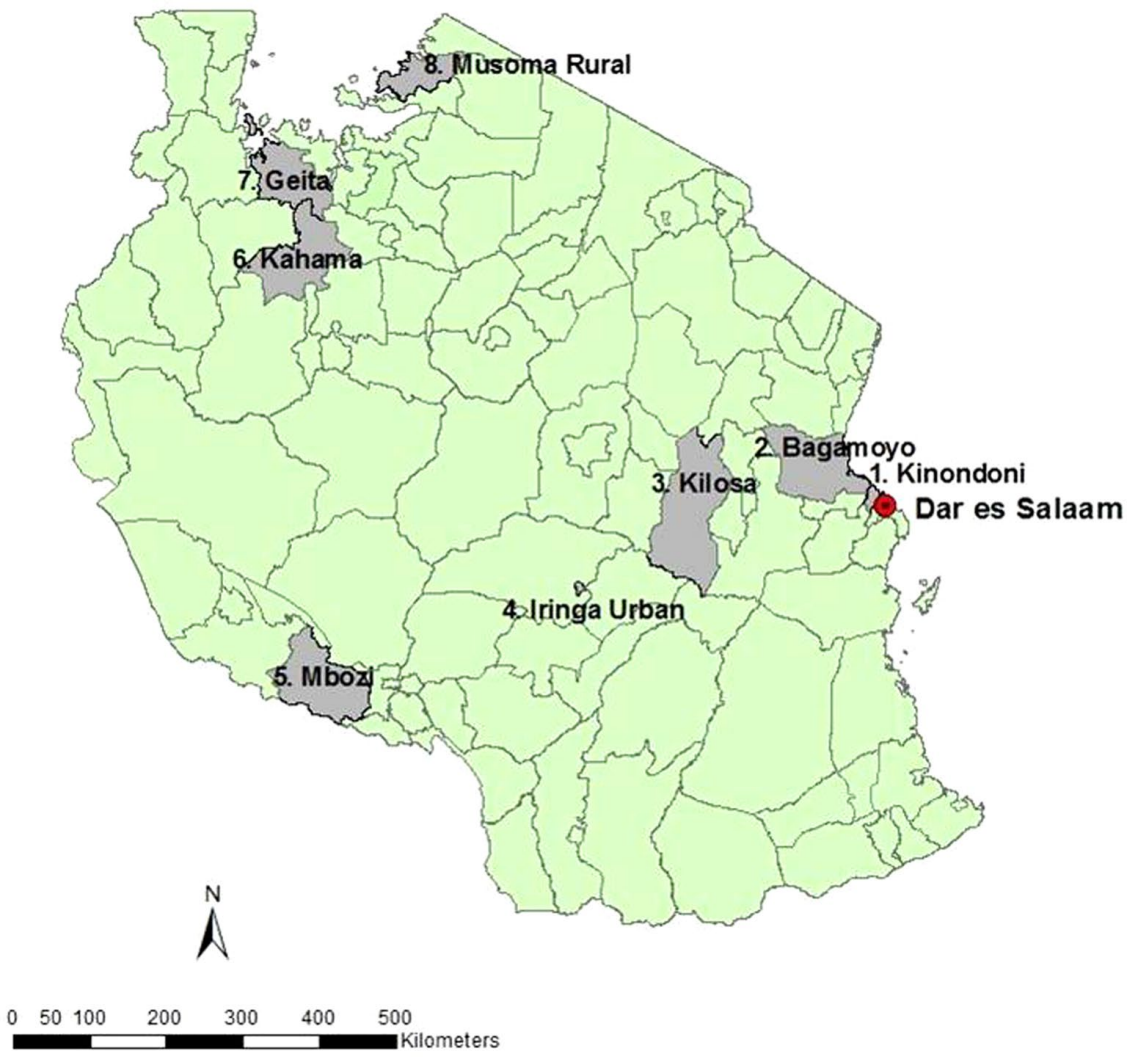
Geographical distribution of eight study districts representing five of eight geographical zones of Tanzania and covering variations in malaria epidemiology and ecology

This study was conducted as part of a long-term project on LLIN durability in Tanzania [16]. The study took place in eight districts (Fig. 1) selected from 23 districts enrolled in the population arm of the sentinel panel of Districts (SPD), sample vital registration with verbal autopsy (SAVVY) [17]. Ten SAVVY villages per district were selected based on their proximity to district council headquarters, except for Kinondoni district where SAVVY only covered six villages. Using SAVVY baseline household information, 45 households per village (3420 households in total) were randomly selected using the ‘sample’ function in the statistical software R 3.1.1. [16].

### Data collection

Cross-sectional household surveys were conducted between October and December 2013. The surveys involved collections of two sets of data. First, household information was collected by a pre-tested semi-structured household questionnaire using Google Nexus tablet computers programmed with the open-source survey tool kit ODK Collect [18]. Information collected included household characteristics (household assets and housing conditions), number of mosquito nets (including Olyset campaign nets) received, any net lost since initial distribution and reasons for losing them. Second, all nets present in the sampled households were collected and replaced with new nets. All collected nets were returned to Bagamoyo Research and Training Centre (BRTC), part of Ifakara Health Institute (IHI), and their colour, size, product and manufacturing date (if available on label) were recorded to establish the total number of campaign nets that were still present in households at the time of the survey. Government Olyset campaign nets could be distinguished from nets from other sources because they were light-blue in colour and of single size (4 × 6 × 7 feet). However, it was not possible to identify which mass campaign the nets originated from (U5CC or UCC) as very few nets retained labels with legible manufacturing dates. Net age was estimated from data on when nets had been distributed to each district during the UCC campaign. This underestimates the age of nets obtained during the U5CC campaign, which took place approximately 12 months before the UCC campaign. This is believed to be a reasonable assumption since the UCC was a considerably bigger campaign distributing 17.6 million nets compared to U5CC’s 7.8 million [4]. From all light-blue single sized Olyset campaign nets collected, 200 nets (25 nets per district) that still had legible manufacturing labels attached were selected for counting holes (for physical integrity) and bio-efficacy testing. All Olyset nets distributed in Tanzania and tested in this study were of the old knitting pattern, which was replaced by a new knitting pattern in 2014.

### LLINs testing procedures

#### Attrition

Net attrition, the inverse of net survivorship, refers here to the proportion of nets distributed to households during the UCC or U5CC campaigns which are no longer in use due to nets either being discarded, used for something else than sleeping under [19] or given away for others to use [8]. It is calculated by dividing the number of nets lost by the number of nets given out to each household. Unfortunately, in this study, it was not possible to establish the number of campaign nets given to each household through official net distribution channels. Therefore, recall information on the number of nets received by each household was collected. Net survivorship was calculated by setting the recalled total number of campaign Olyset nets (U5CC and UCC) received by each sampled household as the denominator, and the total number of light-blue Olyset campaign nets physically collected as the numerator. Net attrition was calculated using the following formula: $\left( {1 - \frac{\text{Total campaign light} \,{-} \,{\text{blue Olyset nets present in the households }}}{\text{Total Campaign nets reported to be received by each household }}} \right)$ × 100 %.

#### Physical integrity

Two hundred Olyset campaign nets were sub-sampled for assessment of their physical integrity and repair status. Each sampled net was mounted onto a 180 cm × 160 cm collapsible net frame. The number of differently sized holes of each sampled net was recorded following WHO guideline [20]. The physical integrity of nets was categorized by the proportionate hole index (pHI), which is calculated as follows: $$ pHI = \left( {size{\rm{ }}\,1{\rm{ }}\,holes{\rm{ }}\,x{\rm{ }}\,1} \right) + \left( {size{\rm{ }}\,2{\rm{ }}\,holes{\rm{ }}\,x{\rm{ }}\,23} \right) + \left( {size{\rm{ }}\,3{\rm{ }}\,holes{\rm{ }}\,x{\rm{ }}\,196} \right) + \left( {size{\rm{ }}\,4{\rm{ }}\,holes{\rm{ }}\,x{\rm{ }}\,578} \right)\ $$

Based on their pHI value, LLINs were assigned to one of the following WHO categories: “good” (pHI ≤64), “damaged” (pHI = 65–642) and “too torn” (pHI ≥643). The first two categories were then combined as “serviceable” while those “too torn” were defined as “unserviceable” nets.

#### Bioassays

After the physical integrity assessment, two squares of netting (25 × 25 cm) were cut from each of four positions on each of the 200 sampled nets following WHO procedures [8]. One netting sample per position per net was tested with cone bioassays as per WHO guidelines [20], the other netting sample was sent for chemical analysis of permethrin content (see below).

Cone assays were carried out at 27 ± 2 °C and 75 ± 10 % relative humidity. Four standard WHO cones were used per netting sample. These cones were laid on the netting sample pinned to a board, held at a 45° angle to prevent mosquitoes from resting on the cone surface. The mosquitoes used were pyrethroid susceptible *Anopheles gambiae* sensu stricto (*s.s*.) aged 3–8 days old originally colonized from wild-caught gravid females in Njage, South-East Tanzania in 1996. Mosquitoes were reared according to standard procedures [21]. Five mosquitoes were introduced into each of the cones and exposed to the netting samples for 3 min, after which they were transferred to holding cups and held for 24 h with access to 10 % sugar solution. One untreated netting sample was used as a control for each net tested. Mosquito knockdown (any mosquito that cannot stand or fly in a coordinated manner) and mortality (mosquitoes that show no movement) were recorded 60 min and 24 h after exposure, respectively.

Net samples that failed cone test cut-off points (i.e. ≤80 % mortality and/or ≤95 % knockdown) were further tested in WHO tunnels assays with *An. gambiaes.s.* Kisumu strain, 3-8 days old at Amani Research Centre (Muheza, Tanzania) using rabbits as bait following WHO guidelines [20]. Of the four squares per net, the square that elicited mosquito mortality closest to the average mortality for the whole net sample was selected and tested in the WHO tunnel. Mosquitoes were scored as alive, dead, blood-fed or unfed. Delayed (24 h) mortality was recorded for the live mosquitoes. Net samples with mortality ≥80 % and/or blood feeding inhibition ≥90 % in tunnel tests were regarded to pass WHO tunnel assay criteria [18]. If mortality in control replicates was between 5–20 %, it was corrected by Abbott’s formula [22]. If control mortality was above 20 %, the whole test was discarded and repeated as per WHO guidelines.

#### Permethrin content

Four netting square samples from each net were individually packed in foil, labelled and stored at 4 °C before being sent for analysis of permethrin content at the WHO Collaborating Centre for Quality Control of Pesticides, Walloon Agricultural Research Centre (CRA-W) [23]. The analytical method used for determination of permethrin in Olyset samples was the CIPAC method 331/LN/M/3. This method involved extraction of permethrin in a water bath (85–90 °C) for 45 min with heptane in presence of triphenyl phosphate as internal standard and determination by Gas Chromatography with Flame Ionisation Detection (GC-FID). The performance of the analytical method was controlled during the analysis of samples in order to validate the analytical results. The results were recorded as either net sample with permethrin content below the lower WHO tolerance limit of 15 g/kg of the target dose of a new net of 20 g/kg ± 25 % [15–25 g/kg].

### Data analysis

Results from WHO cone and tunnel bioassays, insecticide content and physical condition of nets were recorded on standardized forms and double entered in Excel spreadsheet for validation. Cleaning and analysis of data were done using Stata 13.0 statistical software (Stata Corp., College Station, USA).

Socioeconomic status (SES) of each sampled household was assessed by constructing a household wealth index based on household measures that included household assets and housing condition [24, 25]. A weighted sum of the factors and household assets for each sampled household was calculated using principal component analysis (PCA) and the best model was the one with the lowest Akaike Information Criterion (AIC) value [26]. The sample was then divided into wealth quintiles.

Attrition data was analysed by logistic regression with proportion of nets lost as the outcome variable and district as the explanatory variable. Data from the physical integrity assessment were analysed by logistic regression with the binary outcome of proportion of nets in serviceable condition (pHI <643) relative to nets in unserviceable condition (pHI ≥643) and SES wealth quintile, net age in months since UCC distribution grouped into four categories (<25 months group, 25–36 months group, 37–48 months group and >48 months group), number of sleepers per bed, presence of rats, and type of sleeping space set as explanatory variables. A likelihood ratio test was used to compare two models in order to test the significance of particular explanatory variables.

Data from WHO cone and tunnel assays were analysed using logistic regression with the proportion that passed WHO cone or tunnel criteria as outcome variables. Net age categories in months since UCC distribution and net condition by pHI were explanatory variables.

Data from chemical tests were analysed using logistic regression with the proportion of nets exceeding WHO cut-off for permethrin content. Net age categories in months since UCC distribution and net condition by pHI as explanatory variables. Different relationships were also explored in a multivariate analysis model. In all analyses, robust standard errors were used to account for clustering in the data at the district level.

### Ethics

Ethical approval was obtained from the London School of Hygiene and Tropical Medicine (LSHTM—UK) Research Ethics Committee (Reference number 6333), Ifakara Health Institute in Tanzania (Reference number IHI/IRB/No: 19-2013), and the Tanzanian National Institute for Medical Research (Ref: NIMR/HQ/R.8a/Vol. IX/150 and NIMR/HQ/R.8c/Vol. I/285). Before household interviews, the study was explained in Kiswahili or the local language and written informed consent was obtained from each household head or other adults above the age of 18 years. Questionnaires were coded with a unique ID code and names were not taken to ensure confidentiality. All sampled households received free replacement nets for each sleeping space.

## Results

### Demographic characteristics

A total of 3398 households (out of 3420 target households) were sampled in 76 villages in eight districts in Tanzania. From these households, 6529 nets were collected of which 5047 (77 %) were LLINs, which included campaign and non-campaign mosquito nets. The total number of household members was 18,597, with an average household size of 5.7 people (95 % CI 5.6–5.8). Each household had an average of 3.1 (95 % CI 3.0–3.2) sleeping spaces of any type and 2.4 (95 % CI 2.3–2.4) mosquito nets of any kind. Over 66 % (95 % CI 64.9–68.6 %) of the household heads had attained primary school education.

### Net attrition

**Fig. 2:**
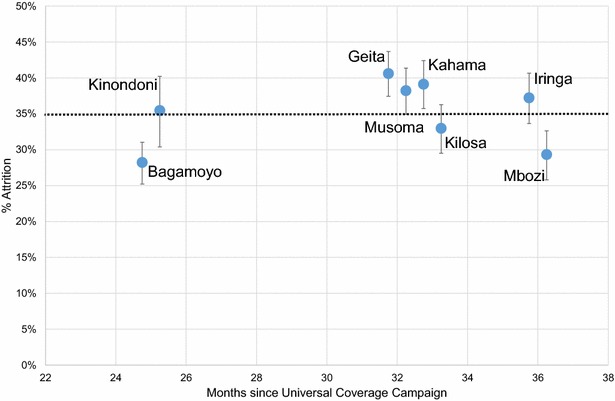
Attrition of Olyset campaign nets by age (month) and districts. The Figure shows the proportion of nets no longer present in the household in each district since initial distribution between 2009 and 2011

Households reported to have received 6067 campaign LLINs between 2009 and 2011. In 2013 during the retrospective survey, 3922 (65 %) light-blue Olyset nets were still present, giving a mean net attrition of 35 % (95 % CI 34.0–37.0 %). Attrition of campaign nets varied significantly between districts (χ^2^ = 54.56, p < 0.001). Bagamoyo and Mbozi lost the fewest campaign nets (28.2; 95 % CI 25.2–31.0 and 29.3 %; 95 % CI 25.8–32.6 %, respectively) whereas the districts around Lake Victoria (Fig. 1) showed the highest attrition (e.g. Geita: 40.6; 95 % CI 37.4–43.7 %; Fig. 2). Age of nets was confounded by district as the campaigns were staggered geographically and temporally, but there was no observed trend of time on net attrition (Fig. 2). Of those nets no longer present, 84 % were reported to have been discarded, 14 % were said to have been given away, sold or stolen and 2 % had been used for alternative purposes such as fencing, screening of doors and windows, fishing and protecting chickens. The reasons given for discarding nets were that they were too torn (94 %), dirty (3 %), or the user did not like them (3 %).

### Net integrity

Out of the 3922 light-blue Olyset nets collected, 200 nets were sampled and their physical integrity was assessed. Two of these were excluded from the analysis because their unique identifying labels were lost, leaving a total of 198 sub-sampled nets.

Overall, more than half of the nets (n = 106; 54 %) were between 25–36 months of age, while 17 nets (10 %) were more than 4 years old (Table 1). Twelve percent (n = 24) had no holes, while 88 % (n = 174) had at least one hole. The frequency distribution of pHI was right-skewed with a median pHI of 279 (Interquartile range—IQR = 9–1220). Based on WHO pHI categories, 37 % (n = 73) were considered in “good” condition, 24 % (n = 47) were “damaged” while 39 % (n = 78) were “too torn”. Following WHO classification [12], 61 % (120/198) of the sampled nets were still in serviceable condition (good and damaged conditions). The only factor that was found to have a significant effect on net integrity was presence of rats in households (Table 2). The presence of rats decreased the odds of having a net in good condition by 60 % (OR = 0.4; 95 % CI 0.1–1.0; p = 0.05). Neither SES wealth quintile, type of sleeping space, number of sleepers under a mosquito net nor net age showed any statistically significant relationship with the physical condition of the net (Table 2). Two-to-four years after the distribution campaigns, 39 % (95 % CI 38–40 %) of nets were functional i.e. 61 % still serviceable of the 65 % campaign nets remaining. Only 17 % of the sampled nets had been observed to be repaired.

**Table 1:** Number and proportion of campaign Olyset nets by age and proportionate Hole Index (pHI)

Time of use since distribution (months)	pHI ≤64 ‘good’ ( %)	65 < pHI ≤642 ‘damaged’ ( %)	pHI ≥ 643 ‘too torn’ ( %)	Total
<25	15 (31)	12 (25)	21 (44)	48
25–36	41 (39)	30 (28)	35 (33)	106
37–48	13 (48)	2 (7)	12 (44)	27
>48	4 (24)	3 (18)	10 (59)	17
Total	73 (37)	47 (24)	78 (39)	198

**Table 2:** Multivariable analysis on factors that might affect the good physical condition (proportionate Hole Index ≤64) of Olyset campaign nets distributed 2–4 years earlier in Tanzania

Explanatory variables	Odds ratio	Likelihood of net being in good physical condition (pHI ≤64)		
		95 % CI	P value	Overall P value (likelihood ratio test)
Socioeconomic status of households				
Least wealthy (1)	1.0			0.88
(2)	0.9	0.4–2.7	0.98	
(3)	0.7	0.3–2.1	0.59	
(4)	1.1	0.4–3.2	0.89	
Wealthiest (5)	1.3	0.5–3.5	0.65	
Net age (months)				
≤25	1.0			0.09
26–36	1.9	0.8–4.2	0.13	
37–48	1.0	0.4–3.1	0.95	
>48	0.6	0.1–2.1	0.39	
Presence of rat faeces/rats in household				
No	1.0			*0.04*
Yes	0.4	0.1–1.0	*0.05*	
Type of sleeping space				
Reed mat	1.0			0.78
Mattress, no frame	0.6	0.1–3.2	0.54	
Bedframe made from sticks	0.7	0.2–2.7	0.56	
Wooden and iron bedframe	0.6	0.2–1.9	0.32	
Number of persons per net				
1 user	1.0			0.44
2 users	0.7	0.3–1.4	0.39	
3 users	0.6	0.2–1.7	0.41	
4 users	3.2	0.3–33.9	0.33	

Italic values indicate significance of p value (p < 0.05)

### Bioassay and permethrin content results of subsample of nets of different age

Results of the bioassay tests and permethrin content analysis of 198 sub-sampled nets of different ages are presented on Table 3. The mean permethrin content was 16.5 g/kg (95 % CI 16.2–16.9 g/kg). Only nets aged 4 years and older had permethrin content 14.0 g/kg (95 % CI 12.0–16.0 g/kg), which is below the WHO recommended concentration for brand-new nets.

**Table 3:** Number and proportion of Olyset nets of different ages with recommended permethrin content and passed WHO cone/tunnel tests criteria

Explanatory variables	Net age (months) ( %)			
	≤25	26–36	37–48	>48
Number of sub-sampled nets	48 (24)	106 (54)	27 (14)	17 (8)
Cone assay				
Proportion of nets passed WHO cone assay criteria^a^	30 (62)	64 (60)	20 (74)	12 (71)
Pass WHO cone or tunnel tests^b^				
Proportion of nets passed WHO cone or tunnel tests criteria	45 (94)	105 (99)	27 (100)	17 (100)
Chemical residue				
Proportion of nets with recommended permethrin content	41 (85)	86 (81)	20 (74)	8 (47)
Mean permethrin content in g/kg (95 % CI)	16.8 (16.3–17.2)	16.8 (16.4–17.4)	16.5 (15.7–17.3)	14.0 (12.0–16.0)

^a^ WHO cone assay criteria: ≥95 % knockdown and/or ≥80 % mortality pass rate

^b^ WHO tunnel test criteria: ≥80 % mortality and/or ≥90 % blood feeding inhibition pass rate

The cone assay data of sampled nets are presented as proportion of nets that passed WHO cone assay criteria, i.e. nets that caused more than 95 % knockdown and/or more than 80 % mortality. The average knockdown was 89.5 % (range 86.7–92.2 %) at 60 min post exposure while average mortality was 55.7 % (range 51.9–59.3 %) at 24 h post exposure with susceptible mosquitoes. Overall 63.6 % (126/198) of nets passed the WHO cone assay criteria. Age of the net had no significant effect on probability of passing cone criteria (Table 4). The 72 nets that failed cone assay criteria were tested in WHO tunnel assays, which is presented as proportion of nets that passed WHO tunnel assay criteria i.e. nets that caused more than 90 % blood feeding inhibition and/or more than 80 % mortality. Ninety-four point three percent (range 89.9–98.8 %) of mosquitoes did not blood feed while average mortality was 97.4 % (range 96.7–98.1 %) at 24 h post exposure. The overall percent of nets passing the tunnel tests criteria was 94.4 % (89.0–99.9 %), which was not statistically explained by net age (Table 4).

**Table 4:** Multivariable logistic regression analysis of the bio-efficacy and permethrin content of sampled Olyset campaign nets

Explanatory variables	Pass cone assay criteria			Above 15 g/kg permethrin		
	Odds ratio	95 % CI	P value	Odds ratio	95 % CI	P value
Net age (in months since initial distribution)						
Old nets (>25)	1			1.0		
Newer Nets (≤25)	0.9	0.5–1.9	0.9	2.1	0.8–5.3	0.11
Physical condition of net						
Net in unserviceable condition	1			1.0		
Net in serviceable condition	2.4	1.3–4.4	*0.04*	4.1	2.0–8.5	*0.001*

Italic values indicate significance of p value (p < 0.05)

### Bioassay and permethrin content results of subsample of nets of different physical condition

Results of the bioassay tests and permethrin content analysis of 198 sub-sample nets of different physical condition (i.e. good, damaged and unserviceable condition) are presented in Table 5. The proportion of nets (of different physical condition) that passed cone assay, tunnel tests and with recommended permethrin content are presented as percentages. Overall, 96 % (116/120) of nets in serviceable condition passed WHO cone or tunnel tests cut off criteria while all nets in unserviceable condition passed WHO cone or tunnel tests cut off criteria.

**Table 5:** Number and proportion of Olyset nets of different physical condition with recommended permethrin content and passed WHO cone/tunnel assay tests criteria

Explanatory variables	Net physical condition (pHI)			
	Good (pHI <64) ( %)	Damaged (pHI = 64–642) ( %)	Serviceable condition** (pHI <643) ( %)	Unserviceable condition (pHI >642) ( %)
Number of subsampled nets	73 (37)	47 (24)	120 (61)	78 (39)
Cone assays				
Proportion of nets passed WHO cone assay criteria^a^	79 (n = 58)	60 (n = 28)	72 (n = 86)	51 (n = 40)
Cone assays or tunnel tests^b^				
Proportion of nets passed WHO cone or tunnel tests criteria	97 (n = 71)	96 (n = 45)	97 (n = 116)	100 (n = 78)
Chemical residue				
Proportion of nets with recommended permethrin content	92 (n = 67)	81 (n = 38)	88 (n = 105)	64 (n = 50)
Mean permethrin content in g/kg (95 % CI)	17.8 (17.4–18.2)	16.5 (15.8–17.2)	17.3 (16.9–17.7)	15.4 (14.7–16.0)

** Serviceable condition include nets in good condition and those in damaged condition

^a^ WHO cone assay criteria: ≥95 % knockdown and/or ≥80 % mortality pass criteria

^b^ WHO tunnel test criteria: ≥80 % mortality and/or ≥90 % blood feeding inhibition pass criteria

A multivariable analysis was conducted to explore different relationships (Table 4). The odds of a net passing cone assay tests was 2.4 times (OR = 2.4, 95 % CI 1.3–4.4, p = 0.04) greater for nets in serviceable condition as compared to nets in unserviceable condition. Permethrin content was four times higher (OR = 4.1, 95 % CI 2.0–8.5, p = 0.001) among nets in serviceable condition as compared to those in unserviceable condition.

## Discussion

This retrospective study in 3398 households in Tanzania found that more than a third of campaign nets had been lost since the government campaigns in 2009 and 2011, and that a further 39 % of the nets had large hole surface areas, leading to an urgent need to replace LLINs in Tanzania.

In this study, attrition of LLINs was higher than that observed in Western Kenya after 5 years [27], but similar to net loss in Rwanda [28] and Nigeria [11]. In Tanzania, net condition (i.e. the number and size of holes) was the primary reason given by study households for discarding of nets, a finding mirrored in a recent multi-country investigation, which found that 63 % of lost nets had been discarded, primarily because they were perceived as too torn (93 %) [19].

More than half of nets were still in a serviceable physical condition, hence theoretically effective in protecting individuals against mosquito bites. Percent of nets with holes observed in this study was higher than in Western Uganda where the majority (87 %) of polyester nets were in serviceable conditions after three and a half years and 23 % of nets had no holes at all after 36–42 months of use [9]. In Zambia, on the other hand, 30 % of polyester and poly-ethylene nets were classed as ‘too torn’ after 30 months in the field [29]. Unlike other studies [13, 26], this study did not find a relationship between net age and its physical condition. This may be because very worn nets are more likely to be discarded, resulting in lower hole counts in older nets as was found in Zambia [30]. The only statistically significant determinant of a net being unserviceable was the presence of rats, a parameter often associated with poor physical condition of mosquito nets [14, 31]. With differences in geographical settings between different study villages, assessing degradation using only 198 nets is likely to have been an insufficient sample size to detect differences between nets of different ages, although it is sufficient to demonstrate that nets do need to be replaced.

The low rates of repair observed in this study have also been observed in Ethiopia [10] and in Kenya [14], suggesting that barriers to net care and repair may exist [32]. Such low repair rates, which probably result in more nets becoming unserviceable, are particularly important because more than two-thirds of the unserviceable nets still contained permethrin concentrations above 15 g/kg—the lowest permethrin threshold set by WHO for brand-new nets. This means that these nets, if repaired, could still provide good individual protection and it is likely that these nets continue to provide community protection by killing mosquitoes [33, 34] or inhibiting blood feeding [35] despite containing holes. However, it should be noted that the nets collected in the households and tested for bio-efficacy are those that have been retained by households, probably precisely because of their better condition and may have been stored for later use.

Despite not being able to plot LLIN survival due to the collection of data during only one time point, this study nevertheless adds a useful data point to the growing table of net durability in various countries and of numerous net products [29, 36]. This study showed a functional LLIN survival of 39 % two-to-four years after the distribution campaigns, which is lower than the median survival of 50 % after 3 years of a ‘three-year net’ [14]. This survival estimate is based on two facts: (a) Hole counts were performed on a sub-sample of 198 nets; and (b) the WHO cut-off criteria of a ‘serviceable’ net is in terms of protection against malaria. The relevance of the hole index as a measure of personal protection is currently lacking hard evidence and requires further investigation [37]. The relative contribution of insecticide and pHI to personal and community protection will be further studied by this study team.

The results from this study could have several implications for the LLIN strategy of the Tanzanian National Malaria Control Programme (NMCP). Firstly, a clear challenge observed from this and other studies was that owners discard nets mostly because they are perceived to be in too torn condition (unserviceable). However, two-thirds of the sampled nets in the unserviceable category were found to have permethrin concentrations above the recommended WHO threshold criteria, which could pose environmental problems when discarded inappropriately [38]. Therefore, the government needs to introduce a better mechanism of collecting and disposing of unserviceable nets to prevent environmental pollution and introduction of insecticides to the environment. As a first step, the manufacturer A–Z Textile Mills Limited recycled all Olyset nets collected by the study team, but a more widely applicable system of net recycling by the government or industry should be developed. A second challenge observed was the functional LLIN survival rate, which fell 11 % short of the expected median survival of a ‘three-year’ net. In addition, target net coverage goals of at least 80 % coverage by 2020 as set by the Tanzanian NMCP will not be maintained through mass campaigns taking place in 3 year cycles. Therefore, continuous net replenishment has been implemented through the TNVS between 2004 and 2014 and through schools in Southern Tanzania since 2013. A new free LLIN distribution mechanism through reproductive and child health clinics will be rolled out in 2016 to replace the TNVS (K. Kramer, *pers. comm.*). Thirdly, given the bio-efficacy and permethrin contents of the collected nets, the government and NMCP need to improve their behavioural change communication strategy so it delivers locally appropriate education messages on net care and repair, which could serve to increase net retention and personal protection and hence prolong the lifespan of LLINs.

A limitation of this study was its retrospective sampling design. Data collection on attrition relied on respondents’ recall information (hence recall bias), because it was not known how many Olyset nets had been distributed to each household. In addition, it was impossible to establish the exact age of nets due to difficulties in distinguishing between U5CC and UCC Olyset nets. Therefore, the attrition rate presented in Fig. 2 is a conservative estimate of the smallest possible age gap. A large prospective study is currently on-going in Tanzania to compare three net brands in the same study households over three years [16], which will be able to capture attrition and physical degradation more precisely and accurately.

## Conclusions

The findings from this study highlight that the functional survival of Olyset nets two-to-four years after campaigns is 39 %, which is below the median survival of a ‘three-year’ net of 50 % as recommended by WHO. Therefore, LLINs are urgently needed in Tanzania to substantially increase access to serviceable mosquito nets. A universal mass campaign is currently ongoing to increase baseline levels, but high coverage must be maintained through continuous distribution mechanisms. When all the measurements of LLINs durability are taken together, it can be concluded that around 65 % of LLINs distributed between 2009 and 2011 were still present in households, and a majority of them had retained target insecticide levels and were biologically effective against anopheline mosquitoes. This means that these nets could still be useful if they were repaired and they may pose environmental problems if incorrectly disposed of. Therefore, it is recommended the implementation of more targeted care and repair campaigns and investigations into means of encouraging net re-use, net recycling and safe disposal.
